# A Matron's Corner

**Published:** 1887-09-17

**Authors:** 


### "A MATRON'S CORNER."

I WAS very glad to see the suggestion of a "Matron's Corner" in your paper. Two questions occur to me at once. What is the best material for nurses' winter dresses in a very cold locality? Why won't educated women, instead of rushing into nursing, become hospital cooks? A RESTING MATRON.

